# Current Techniques for Inorganic Polyphosphate Detection and Characterisation

**DOI:** 10.3390/biom16081138

**Published:** 2026-08-05

**Authors:** Johanna G. Rodríguez, Thomas Renné

**Affiliations:** 1 Institute of Clinical Chemistry and Laboratory Medicine, University Medical Center Hamburg-Eppendorf, Martinistraße 52, D-20246 Hamburg, Germany; t.renne@uke.de; 2 Irish Centre for Vascular Biology, School of Pharmacy and Biomolecular Sciences, Royal College of Surgeons in Ireland, D02 YN77 Dublin, Ireland

**Keywords:** polyphosphate, polyP detection, fluorescent dyes, recombinant polyP-binding probes, chromogenic and fluorogenic assays, biophysical techniques, biomarker

## Abstract

Polyphosphate (polyP) is an evolutionarily conserved linear polymer of orthophosphate residues with diverse functions across organisms from bacteria to mammals. In addition to its roles in phosphate and energy storage, polyP has been implicated in thrombosis, inflammation, cancer, metabolism, cytoskeletal regulation, and neurodegenerative diseases. PolyP also acts as a molecular scaffold interacting with lysine-rich proteins and may contribute to protein folding and amyloid formation. However, biochemical heterogeneity, including variation in chain-length, subcellular localisation, and supramolecular organisation together with the absence of clearly defined mammalian biosynthetic pathways, has limited mechanistic understanding of polyP biology. Reliable detection and quantification remain challenging because current methods often suffer from limited specificity, chain length bias, insufficient quantitative robustness, and interference from other highly anionic biomolecules such as DNA or RNA. This review summarises current methodologies for polyP detection and characterisation. Emerging polyP-specific probes, including recombinant polyP-binding domains derived from polyphosphatases and conserved histidine α-helical domains may improve qualitative and quantitative analysis of polyP in complex biological systems. Improved analytical strategies will be essential to define the physiological roles of polyP and evaluate its potential as a biomarker and therapeutic target.

## 1. Introduction

Polyphosphate (polyP) is an evolutionarily conserved linear polymer of orthophosphate (Pi) residues linked by high-energy phosphoanhydride bonds [1,2]. While well-characterised in microorganisms as in energy and phosphate storage [3], its precise biochemical synthesis, distribution, and metabolism in mammalian systems remain poorly understood [4]. This knowledge gap is explained by the absence of mammalian homologues to bacterial polyphosphate kinase (PPK) or yeast vacuolar transporter chaperone 4 (VTC4) complexes, which act as a transmembrane polymerase generating polyP, as well as the lack of clear exopolyphosphatases (PPX), which complicates the genetic manipulation and quantitative analysis of intracellular polyP [5,6,7]. Although mitochondria, inositol phosphate metabolism, and non-specific phosphatases are implicated in polyP dynamics, the absence of specific polyP metabolizing enzymes has prevented selective manipulation of intracellular polyP and the establishment of standardised analytical methods in mammalian cells and tissues [8,9,10,11,12].

Biochemically, polyP is highly heterogeneous with respect to chain length, cellular localisation, and molecular interactions [13]. PolyP chain lengths range from a few residues to thousands depending on the organism and cellular niche [14]. For instance, mammalian platelets secrete medium-chain polymers (80–100 residues) but mostly contain long-chain polymers (>300 P_i_ residues) intracellularly that are exposed on the plasma membrane upon activation [15,16,17]. Because its backbone is highly negatively charged, polyP preferentially interacts electrostatically with cations and proteins [18], acting as a protein-stabilising chaperone [19]. PolyP also accumulates within the nucleolus [20]. Whether polyP modifies proteins covalently via polyacidic, serine-, and lysine-rich (PASK) domains or acts strictly through non-covalent, ionic interactions remains an active debate [21,22,23].

PolyP has been detected in multiple mammalian tissues and subcellular compartments, including platelets [24,25,26], with functions extending far beyond its classical role as an energy and phosphate reservoir [27]. In thrombosis, platelet-derived polyP has emerged as an important mediator that drives thrombus propagation and triggers inflammation [28,29]. PolyP activates factor XII in vivo, triggering the thromboinflammatory plasma contact system [30]. In vitro, polyP additionally accelerates thrombin generation, modulates fibrin clot structure, and influences fibrinolysis [31,32,33,34]. PolyP has also been associated with inflammatory signalling in endothelial cells, where extracellular polyP activates proinflammatory pathways through P2Y1 receptors and the receptor for advanced glycation end products (RAGE) [35].

Beyond thromboinflammation, polyP is increasingly implicated in diverse cellular and pathological processes, such as mast cell biology and tumour-associated inflammation [32]. In colorectal cancer, intracellular polyP has been detected in subsets of CD68-expressing tumour-associated mast cells [36]. Overall, these findings have generated considerable interest in polyP as both a potential biomarker and a therapeutic target in cancer and thromboinflammatory diseases.

Furthermore, polyP functions as a molecular scaffold by interacting with proteins enriched in lysine-rich domains [22,23]. A recent large-scale screen of recombinant human proteins expressed in *E. coli* demonstrated that these lysine-rich regions frequently exhibit polyP-dependent electrophoretic mobility shifts [22,23]. This is particularly evident among nuclear and RNA-binding proteins, where polyP is linked to the regulation of ribosomal RNA transcription and RNA polymerase I activity, suggesting broader roles in transcriptional control and chromatin organisation [37]. Beyond the nucleus, polyP participates in ATP-dependent processes that regulate actin cytoskeleton dynamics and actomyosin activity [38]. Additionally, in vitro studies show that polyP acts as a molecular chaperone for unfolded proteins, promoting stable β-sheet-rich conformations and modulating oligomeric intermediates during amyloidogenesis [39,40,41]. These observations position polyP among the few endogenous molecules capable of regulating amyloid formation and tau aggregation, linking it closely to neurodegenerative disorders [42,43].

The growing interest in polyP across thrombosis, inflammation, cancer, metabolism, and neurodegeneration highlights the need for robust analytical platforms capable of accurately detecting and characterising this polymer in biological systems. Improved analytical and diagnostic approaches will be essential for defining the physiological roles of polyP, understanding its specific functions, and evaluating its clinical potential. Despite this growing interest, our understanding of polyP biology remains incomplete and limited by methodological constraints. To address this, our review integrates current knowledge on polyP detection techniques, highlighting emerging approaches. Here, we critically compare current methods for polyP localisation, quantification and structural characterisation, with emphasis on assay specificity, chain-length bias, biological matrix interference and essential validation controls.

## 2. Fluorescent Dyes for polyP Detection

### 2.1. Classical Metachromatic Dyes

#### 2.1.1. DAPI and Toluidine Blue

4′,6-Diamidino-2-phenylindole (DAPI) is one of the most widely implemented chemical probes for polyP detection, spanning both in situ spatial imaging and gel-based profiling applications [44]. In fluorescence microscopy, DAPI is highly compatible with both fixed- and live-cell imaging, allowing the localisation of densely packed intracellular polyP aggregates within polyP-rich organelles [45]. Beyond microscopy, DAPI serves as a critical staining agent for polyP size determination following agarose or urea–polyacrylamide gel electrophoresis (PAGE) [46,47]. In these methods, polyP resolves not as discrete bands, but as heterogeneous smears that reflect endogenous length polydispersity; these migration profiles are calibrated against DNA or synthetic polyP ladders to estimate apparent chain lengths [48].

DAPI binds polyP and following binding undergoes a characteristic fluorescence emission shift from blue (450–475 nm) to yellow–green fluorescence (520–550 nm), and it is this shift, rather than a simple increase in intensity, that is used diagnostically to distinguish polyP from other cellular components [49]. However, DAPI has limited sensitivity for short-chain polyP and does not reliably detect polymers below than approximately 15 phosphate residues [49]. DAPI also binds nucleic acids (DNA and RNA) with high affinity, particularly in mitochondria and nuclei or in samples containing extracellular nucleic acids [50]. Because DAPI fluorescence can be influenced by nucleic acids and other phosphate-rich metabolites, DAPI-based signals should be interpreted only in combination with enzymatic degradation, extraction controls, and orthogonal detection methods.

Toluidine Blue O (TBO) is a classical metachromatic dye used for polyP detection in electrophoretic applications. Upon binding to polyP, TBO exhibits a metachromatic shift that allows visualisation of polyP species in gels approximately at 530 nm, producing a purple/red colour [51]. Whereas DAPI is comparatively weak at detecting short polyP chains, TBO performs well for resolving short- to medium-chain polyP species within gel matrices [48]. TBO cross-reacts with intracellular polyanions, nucleic acids including DNA and RNA, and sulphated glycosaminoglycans [52]. Nevertheless, both DAPI and TBO remain core laboratory methods to detect polyP. DAPI is useful for gel-based screening but should not be used as a stand-alone polyP-specific probe in nucleic-acid-rich samples.

#### 2.1.2. Additional polyP Stains

Several alternative classical dyes have been explored for polyP or inorganic phosphorus derivative localisation, although they generally have low analyte selectivity.

Acridine Orange (AO): This metachromatic dye binds to phosphate-rich structures but exhibits poor specificity for polyP due to strong intercalative and electrostatic interactions with single- and double-stranded nucleic acids [53]. Consequently, its polyP applications have therefore been restricted to niche contexts where nucleic acid background can be controlled for or is not present in the same compartment, co-staining of DAPI and Acridine Orange has been used to localise polyP within granules present in sea urchin eggs [54]. In a different application, AO has been used to indirectly track inorganic pyrophosphate turnover by measuring dye uptake in permeabilised procyclic trypomastigotes of *Trypanosoma brucei* [55] and promastigotes of *Leishmania donovani* [56].

Methyl Green-Ammonium Molybdate: This dye does not stain polyP directly (see paragraph 4. below); rather, it is designed to react with orthophosphate degradation products released once the polymer is enzymatically hydrolysed [57]. When combined with polyP-degrading enzymes, the resulting reduced phosphomolybdate complex stains the localised degradation products, allowing resolved polyP bands to be tracked and visualised via PAGE [57].

Nile Red: Primarily used as a lipophilic stain used for intracellular lipid droplets and microplastic detection, and it has found only a niche application in environmental phosphate research, where it is used to evaluate lipid–polyP metabolic shifts under nutrient stress rather than to detect polyP directly [58].

### 2.2. Next-Generation Synthetic Fluorophores: JC-D7 and JC-D8

Compared with DAPI or TBO, the novel fluorescent probes JC-D7 and JC-D8 represent a significant milestone, offering improved selectivity for polyP and allow localisation within living cells and complex tissue architectures due to reduced interference from nucleic acids [59]. The benzimidazolium-derived dyes were discovered through the screening of combinatorial chemical libraries originally designed to target highly charged polyanions, such as heparin and guanosine triphosphate (GTP) [60,61].

Previous work validated JC-D7/JC-D8 selectivity, demonstrating high-contrast visualisation of intracellular polyP-containing structures by fluorescence microscopy in primary neuronal and astrocytic cultures, *Drosophila* brain tissue, and cellular models of Parkinson’s disease [59].

Furthermore, JC-D7 has successfully localised polyP stores inside specialised, dense organelles, such as the acidocalcisomes of *Chlamydomonas reinhardtii* [62], as well as dense polyP granules within fixed and living plant and algal cells, supporting its utility for detection of long-chain polyP stores [63,64]. However, while JC-D7 and JC-D8 offer selective advantages, specific experimental protocol must be carefully optimised as dye performance and loading kinetics can vary according to experimental conditions and cellular microenvironments [65].

### 2.3. Comparative and Indirect Staining Strategies: SYTOX Dyes

Cyanine-based nucleic acid stains, specifically SYTOX Green and SYTOX Orange are increasingly incorporated into polyP workflows for indirect or comparative analysis. SYTOX dyes should not be regarded as direct polyP probes; their value lies mainly in exclusion or co-localisation strategies that help distinguish extracellular nucleic acids from putative polyP-rich structures. Because SYTOX dyes are net-positively charged and membrane-impermeable, they are traditionally used to label extracellular DNA or to assess membrane integrity during cellular processes like formation of neutrophil extracellular traps (NETs), bacterial death, apoptosis, and necrosis [66,67,68].

The same feature that makes SYTOX dyes useful nucleic acid markers, reintroduces the cross-reactivity problem in a different form. SYTOX dyes also cross-react with extracellular polyP, such as polymers secreted by activated platelets, because the regularly spaced negative charges on the polyP chain mimic the electrostatic and spatial topology of the sugar-phosphate backbone of DNA [69].

To isolate the polyP-specific signal from background nucleic acids, researchers can achieve spatial localisation by combining DAPI, an exclusion dye (SYTOX), and compartment- or protein-specific immunofluorescent markers in the same sample [70]. This work showed that polyP exposed on the platelet surface after degranulation could be localised by co-staining with DAPI, SYTOX Orange, and anti-CD6, using the convergence of all three signals to identify genuine poly-rich regions rather than relying on any single dye [70].

The classifications, chemical structures, applications, limitations, and advantages of the dyes discussed above are summarised in Table 1.

## 3. Protein-Based polyP Probes

### 3.1. Catalytically Inactive or Truncated PPX-Derived Binding Probes

The development of recombinant polyP probes marks a significant advancement in analytical precision, transitioning the field away from non-specific chemical dyes towards protein-based detection tools.

The *E. coli* PPX enzyme is a dimer composed of two N-terminal domains that harbour the enzymatic activity and hydrolyses polyP. Furthermore, two C-terminal domains that mediate substrate binding. PPX deletion mutant PPX_Δ12 lacks the enzymatic domains 1 and 2 and maintains the polyP binding sites. PPX_Δ12 also termed polyphosphate binding domains (PPBD) specifically recognises and binds polyP chains longer than 35 phosphate residues [74,75]. Early validation of this approach was demonstrated in yeast *Saccharomyces cerevisiae*, where antibody-labelled PPX_Δ12 localised pools of endogenous long-chain polyP [76].

Subsequent studies expanded the PPX_Δ12 methodology to map intracellular polyP granules across highly diverse eukaryotic systems, including *Trypanosoma cruzi*, *Trypanosoma brucei*, and *Drosophila* [77,78]. To map human organelle dynamics specifically, a specialised protocol employed a PPX_Δ12 probe carrying an Xpress epitope-tag, an eight amino acid sequence (DLYDDDDK), achieving high-contrast nuclear immunolocalisation and quantification within SH-SY5Y, HeLa, A549, and HEK293 cell lines [79].

PPX_Δ12, has limited interference with nucleic acids and other polyanions such as high molecular weight dextran sulfate, glycosaminoglycans, DNA, RNA, and oversulfated chondroitin sulfate, precisely the panel of interferents that limited the dyes discussed above [80].

Previous studies produced fluorescently labelled PPX_Δ12 probes covalently coupled to the amine-reactive Alexa488 [80]. The PPX_Δ12-Alexa488 probe was subsequently applied in flow cytometry to quantify platelet-associated polyP in human plasma samples [81]. In contrast flow cytometry using polyP binding low molecular dyes e.g., DAPI and JC-D7 enables analysis of large cell populations and dynamic monitoring of polyP-associated responses [82].

As compared to classical metachromatic dyes, like DAPI or Toluidine Blue, recombinant PPX_Δ12/PPBD-based probes provide improved specificity for long-chain polyP [81]. Thus, recombinant polyP probes provide a powerful and reliable probe for visualisation and quantification of the polymer when it is labelled with fluorescent antibodies.

### 3.2. Conserved Histidine α-Helical Domain (CHAD) Proteins

CHAD-containing proteins represent a second, structurally distinct class of polyP-binding proteins used for detection [83]. CHAD domains form stable interactions with polyP granules, and these domains have been proposed as highly selective polyP recognition modules [84]. Structurally, CHAD proteins typically contain a C-terminal CHAD domain mediating polyP-binding and, in some proteins, an N-terminal CYTH domain associated with nucleotide and phosphate metabolism [84]. Unlike the PPX-derived PPX_Δ12/PPBD, CHAD domains consist of conserved α-helical bundles that form positively charged surfaces and pores, interacting electrostatically with negatively charged polyP chains [83]. The *E. coli* ygiF polyphosphatase, a triphosphate tunnel metalloenzyme, uses its CHAD domain to recognise polyP via similar short sequences at the N-terminus (C-LHTSAR) and the C-terminus (LHSGKR), suggesting that a minimum conserved sequence motif, rather than the whole domain fold, may be sufficient for polyP recognition [85].

Biochemical studies demonstrated that CHAD domains from different evolutionary lineages (e.g., bacteria, archaea, eukaryota) exhibit different binding kinetics and affinities for polyP; for example, CHAD proteins display simple one-to-one binding behaviour, whereas others exhibit multiple affinities [83]. Fluorescently tagged CHAD constructs, including GFP-tagged probes derived from *Saccharolobus solfataricus* or *E. coli* proteins, have been successfully used for polyP localisation [85].

CHAD-based probes offer high affinity and selective polyP binding, and compatibility with live-cell imaging [83,84]. Recombinant polyP-binding probes, such as CHAD and PPX_Δ12/PPBD, represent promising alternatives to classical dye-based detection methods and may improve specifically live-cell imaging, and quantitative analysis of polyP in complex biological systems. The protein structures, biochemical application, limitations, and advantages of PPX_Δ12/PPBD and CHAD are summarised comparatively in Table 2.

## 4. Chromogenic Assays and Fluorogenic Substrates for polyP Quantification

These assays do not measure polyP directly. They quantify orthophosphate released after hydrolysis and therefore require rigorous correction for pre-existing Pi, incomplete hydrolysis, sample matrix effects, and extraction recovery.

### 4.1. Phosphomolybdate Assays

PolyP can be quantified by chemical or enzymatic hydrolysis into Pi, followed by reaction with molybdate under acidic conditions to form a phosphomolybdate complex. Although spectrophotometric and fluorometric quantitative polyP assays share this fundamental chemical principle, they differ in their downstream optical detection strategies.

#### 4.1.1. Malachite Green Assay

The Malachite Green assay is a widely used colorimetric method for the detection and quantification of orthophosphate because of its simplicity, low cost, and high analytical sensitivity [89,90].

The underlying mechanism relies on a two-step reaction under acidic conditions. First, orthophosphate reacts with molybdate to form a yellow phosphomolybdate complex. Rather than undergoing chemical reduction, this complex is directly bound by the triphenylmethane dye (Malachite Green dye), producing a green-coloured complex [91,92]. The intensity of this colour is measured spectrophotometrically at 620–660 nm and absorbance is directly proportional to the orthophosphate concentration in the sample [91,92]. Colour development reflecting complex formation typically requires 10 to 30 min, and commercial reagents generally remain stable for at least 24 h at room temperature, facilitating rapid and high-throughput measurements [93].

Because the Malachite Green reagent reacts with free orthophosphate, polyP must undergo hydrolysis to release measurable monomeric phosphate residues prior to analysis [94,95]. PolyP concentrations are subsequently determined by correlating the absorbance values against a standard curve generated with known phosphate concentrations [96].

Consequently, a Malachite Green assay quantifies total phosphate released from polyP after hydrolysis but is insensitive for information regarding the native polymer chain length and molecular weight distribution [97]. Additionally, the assay is susceptible to several interferences. Arsenates act as structural analogues to phosphate, forming similar complexes that accelerate colour development and cause false-positive results [89].

#### 4.1.2. Molybdenum Blue Assay

The Molybdenum Blue assay is a classical colorimetric method for orthophosphate quantification that relies on a two-step reaction process. Reaction is started with the interaction of orthophosphate and ammonium molybdate under acidic conditions to yield a colourless phosphomolybdate complex [98]. This complex is subsequently reduced by a reducing agent, typically ascorbic acid or stannous chloride, both of which must be prepared fresh daily to generate a stable, intensely blue compound known as phosphomolybdenum blue [99]. The absorbance of this blue complex is monitored within the 700–890 nm range, with the signal intensity directly proportional to the orthophosphate concentration [100].

Similar to the Malachite Green assay, polyP cannot be detected directly by the Molybdenum Blue reagent and requires preliminary acid or enzymatic hydrolysis [101]. Consequently, polyP content is typically quantified indirectly by subtracting baseline free orthophosphate levels from the total phosphate measured after hydrolysis [102]. While the assay is inexpensive, robust, and easy to implement, requiring roughly 15 to 30 min for colour development, its sensitivity is lower than that of the Malachite Green method [99]. The primary limitation of the Molybdenum Blue method lies in its sensitivity to chemical interferences [103].

Arsenates, silicates, and organophosphonates exhibit similar chemical behaviour as orthophosphate under these assay conditions, forming analogous-coloured complexes that lead to false-positive reactions and an overestimation of the true phosphate content [104]. Ultimately, the selection between the Molybdenum Blue and Malachite Green assays for polyP quantification depends primarily on the specific application and sample type.

### 4.2. End-Labelling and Functionalisation

To monitor polyP-binding, hydrolysis and phosphatase activity in real time and to identify polyP interaction partners, recent advances have moved towards the development of covalently modified, end-labelled polyP derivatives that function as fluorogenic polyP substrates [105,106,107]. Two primary synthetic strategies are the synthesis of terminal phenolic esters and the creation of phosphoamidate linkages [105]. Chromogenic and fluorogenic polyP substrates were generated by coupling 4-nitrophenol or 4-methylumbelliferone to the terminal phosphates of polyP via EDAC-mediated phosphoester bonds and doubly end-labeled substrate was used in a two-stage assay in which endopolyphosphatase cleavage exposes new ends for exopolyphosphatase-driven dye release, thereby identifying Nudt2 as an endopolyphosphatase [108].

Furthermore, chemical functionalisation targeting terminal monophosphates allows polyP probes to be synthesised without altering internal polyanionic backbone interactions. Previous work, utilised EDAC-mediated carbodiimide chemistry (1-ethyl-3-[3-dimethylamino-propyl]-carbodiimide) to couple primary amines, fluorophores, or biotin tags to the terminal monophosphate residues of polyP [105]. While this approach facilitated the immobilisation or tracking of polyP without disrupting its polyanionic interactions, the application of these end-labelled fluorogenic substrates remains challenging due to technically demanding synthesis, variable labelling efficiencies, and dependence on polyP chain length [108].

The different classifications, analytical applications, limitations, and advantages of these chromogenic assays and fluorogenic substrates for polyP quantification are summarised in Table 3.

## 5. Advanced Biophysical Techniques for polyP Structure Determination

### 5.1. ^31^P Nuclear Magnetic Resonance (^31^P NMR) Spectroscopy

Phosphorus-31 Nuclear Magnetic Resonance (^31^P NMR) spectroscopy is a powerful non-destructive biophysical tool for resolving the structural characteristics, chain lengths, and compartment-specific phosphometabolic profiles of polyP in biological samples [109]. Based on chemical-shift differences among chemically non-equivalent phosphorus atoms, ^31^P NMR can distinguish orthophosphate, pyrophosphate, and polyP polymers [110,111]. A major advantage of this approach is its capacity for in vivo and compartment-specific measurements within complex systems, allowing the method to be extensively used to study polyP dynamics across diverse biological models [112], including algae, coral tissues, mammalian cell lines, and human platelets [110,113,114,115]. Notably, in human platelets, ^31^P NMR structural studies established that secreted polyP exists in the supernatant as a relatively short chain with a narrow fraction averaging 60–80 phosphate residues, contrasting with the long-chain polymers (exceeding hundreds of residues) found intracellular in platelets and in microbial systems [24,116].

Despite its analytical power, standard solution-state ^31^P NMR cannot detect polyP that is densely packed into insoluble acidocalcisomes, highly condensed, or otherwise immobilised within granules, as the restricted molecular mobility within these microenvironments causes severe dipolar interactions and peak broadening [71]. Furthermore, because polyP samples extracted from biological tissues are inherently polydisperse, the calculated chain lengths typically reflect the collective average properties of a heterogeneous mixture, rather than identifying discrete, individual polymer populations [109,110,111,112,113,115,117]. Finally, the intrinsically low gyromagnetic ratio of the ^31^P nucleus results in low mass sensitivity, requiring high sample concentrations, prolonged acquisition times, and specialised high-field instrumentation that restricts its utility in routine clinical diagnostics [109,112].

### 5.2. Chromatography

Complementing magnetic resonance, chromatographic techniques represent robust methodologies for separating polyP species based on their molecular size and net negative charge properties [118,119,120,121,122]. High-performance liquid chromatography (HPLC) variants, primarily Anion-Exchange chromatography and size-exclusion chromatography, provide high analytical resolution for resolving complex phosphate-containing mixtures and mapping polyP chain-length distributions [123]. However, these approaches require destructive, multi-step extraction and purification protocols prior to analysis, thereby limiting their use strictly to in vitro biochemical applications rather than in vivo or in situ tracking [113,117]. Furthermore, analysis of highly heterogeneous, polydisperse biological polyP pools remain technically challenging due to limited mass sensitivity, demanding sample preparation, and stationary-phase interaction artefacts when resolving highly charged, long-chain polymers [122,123,124].

### 5.3. Mass Spectrometry

Mass spectrometry (MS)-based approaches are also increasingly being adapted for polyP analysis [125]; however, a clear distinction must be made between the direct profiling of the polymer itself versus the characterisation of its cellular interactome. Direct mass spectrometric analysis of intact polyP molecules remains a technical challenge because its highly polyanionic and chemically polydisperse backbone is prone to severe in-source decay, thermal degradation, and unpredictable fragmentation during standard soft ionisation processes like electrospray ionisation (ESI) [71]. Consequently, structural and quantitative MS strategies for this analyte often rely on indirect workflows, such as enzymatic pre-digestion to uniform monomers, terminal chemical derivatisation, or the assessment of total phosphate [124]. Nevertheless, specialised ESI-MS protocols have also been developed to directly characterise and quantify inorganic polyP [126]. Beyond direct polymer analysis, mass spectrometry provides critical insights into polyP protein dynamics, while covalent protein polyphosphorylation and pyrophosphorylation remain mechanistically debated and require validation, recent advances in LC-MS/MS phosphoproteomics have enabled the detection of endogenous pyrophosphosites across human proteins [127], whereas inorganic phosphate detection methods such as ion chromatography inductively coupled plasma (IC-ICP)-MS provide baseline techniques for quantifying free phosphate species [128].

### 5.4. Raman and Infrared Vibrational Spectroscopy

Finally, Raman spectroscopy and Fourier-transform infrared (FTIR) spectroscopy have emerged as powerful, complementary biophysical techniques for polyP characterisation based on characteristic phosphate vibrational signatures, providing spatial information while avoiding fluorescent staining artefacts [129]. Raman-based micro-spectroscopy is particularly advantageous due to its high spatial resolution, which allows for the non-destructive, in situ visualisation of condensed polyP within intracellular microcompartments such as acidocalcisomes or microbial granules [130]. However, polyP detection remains challenging due to low baseline sensitivity and significant spectral overlap with other phosphate-containing compounds [131].

The classifications, applications, limitations, and advantages of these advanced biophysical techniques for polyP structural determination are summarised in Table 4.

## 6. Summary of Analytical Strategies

**Figure 1:**
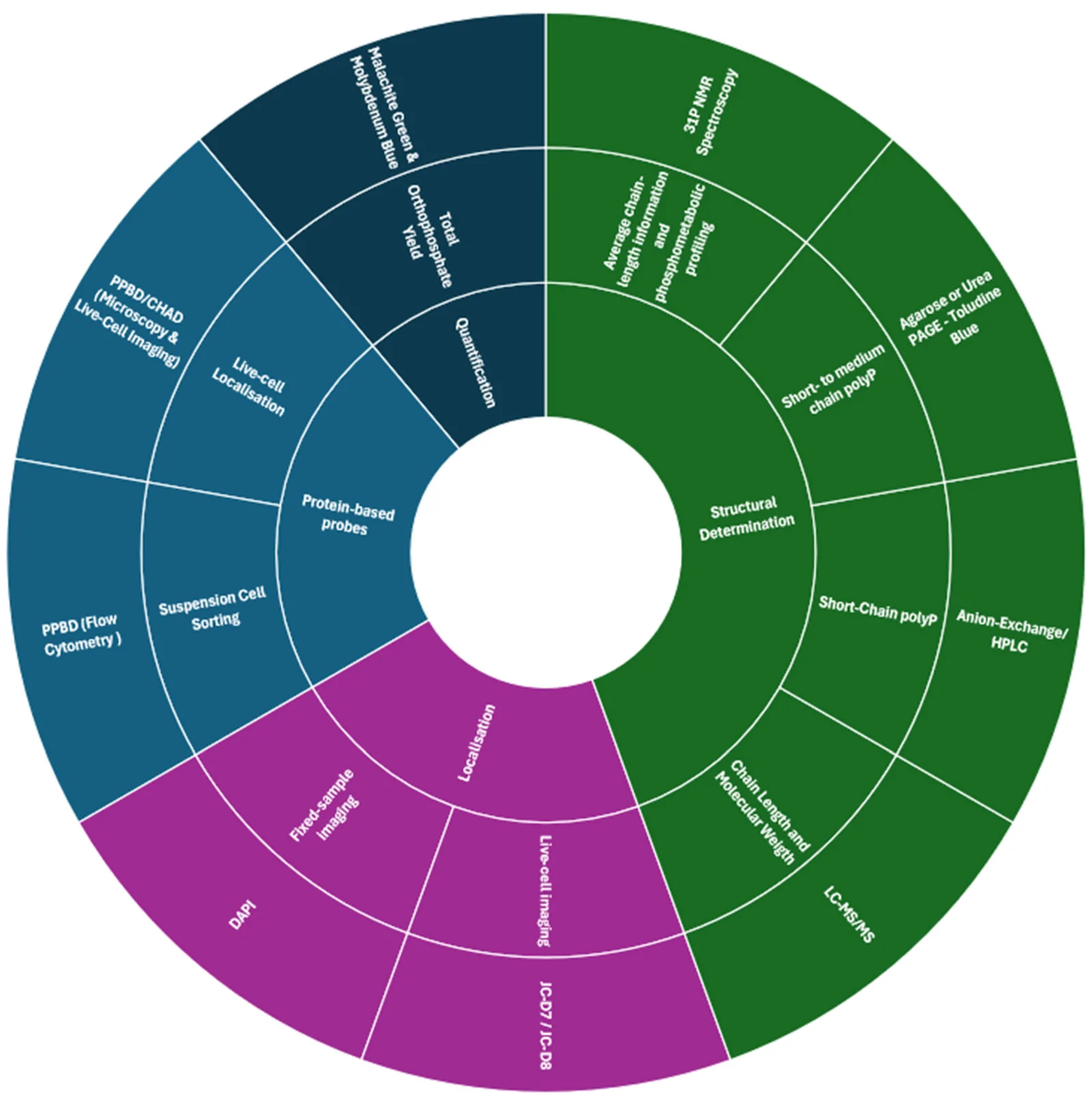
**Comprehensive framework for polyP characterisation and detection methodology.** The classification system is organised into a concentric, multi-layered sunburst hierarchy based on how each method interacts with polyP. The **Inner Ring** divides the analytical toolkit in Protein-Based Probes, Localisation, Structural Determination, and Quantification. The **Middle Ring** maps these approaches to their precise physiological and structural applications, including average chain information and phosphometabolic profiling, short-to-medium-chain polyP, chain length and molecular weight, live-cell imaging, and total orthophosphate yield. The **Outer Ring** shows the specific, actionable techniques available to detect polyP (e.g., Malachite Green, Molybdenum Blue, Phosphorus-31 Nuclear Magnetic Resonance (^31^P NMR) spectroscopy, agarose or urea–polyacrylamide gel electrophoresis (PAGE)-Toluidine Blue, Anion-Exchange, high-performance liquid chromatography (HPLC), LC/MS-MS (liquid chromatography-tandem mass spectrometry), JC-D7/JC-D8, polyphosphate-binding domains (PPX_Δ12/PPBD), conserved histidine α-helical domain (CHAD).

Overall, advanced biophysical and analytical techniques have significantly enhanced our capability to detect and characterise polyP. However, because no single method can overcome every analytical challenge, combining different complementary approaches is essential for reliable results. A conceptual workflow guide, not a strict classification of specificity or performance for method selection is illustrated in Figure 1 and Table 5.

## 7. Conclusions and Future Perspectives

PolyP regulates diverse biological processes through electrostatic interactions with proteins, cations, nucleic acids and other cellular structures. However, its analysis remains challenging because polyP is chemically heterogeneous, chain-length-dependent, highly anionic and often associated with complex biological matrices. No currently available method provides complete information on abundance, chain length, localisation and molecular organisation. Reliable polyP analysis therefore requires orthogonal workflows that combine selective probes, enzymatic controls, quantitative phosphate measurements and structural methods. Future progress will depend on improved recombinant probes, standardised extraction and recovery protocols, and live-cell-compatible approaches that distinguish polyP from other polyanionic biomolecules under physiological conditions.

## Figures and Tables

**Table 1:** Fluorescent dyes for polyP detection.

Dye	Chemical Structure	Applications	Limitations	Advantages
**DAPI**	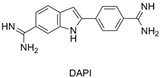	- Fluorescence microscopy [45,71]. - Agarose gel electrophoresis [46]. - Urea–polyacrylamide gel electrophoresis (urea-PAGE) [46].	- High binding affinity to nucleic acids [72]. - Limited sensitivity for short-chain polyP species (<15 residues) [73].	- Simple, inexpensive and highly reproducible workflows [48].
**Toludine** **Blue**	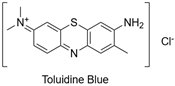	- Urea–polyacrylamide gel electrophoresis (urea-PAGE) [46]. - Agarose gel electrophoresis [46].	- High binding affinity to nucleic acids [72].	- Simple, inexpensive and highly reproducible workflows [48]. - Performs well for short- to medium-chain polyP tracking [48].
**JC-D7/** **JC-D8**	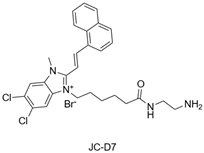	- High-resolution fluorescence microscopy [59].	- Optimal protocols and loading kinetics vary according to experimental conditions [65].	- Improved specificity for polyP localisation in living cell. - Reduced background interference from nucleic acids [59].
**SYTOX dyes**	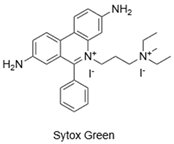 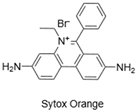	- Multi-spectral fluorescence boundary exclusion microscopy [70].	- Total lack of intrinsic selectivity for polyP directly [69,70]. - Requires complex indirect boundary co-staining workflows [70].	- High affinity for nucleic acids allows precise multi-colour spatial boundary exclusion [66].

**Table 2:** Protein-based polyP probes.

Protein Structure of the Probe	Applications	Limitations	Advantages
**PPX_Δ12/PPBD** 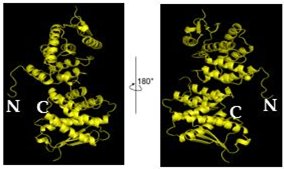	- Fluorescence microscopy [76]. - Flow cytometry [81].	- Requires recombinant protein expression and demanding isolation purification workflows [80,81].	- Improved specificity for long-chain polyP detection [81]. - Reduced interference from nucleic acids and other polyanions [80].
**CHAD** 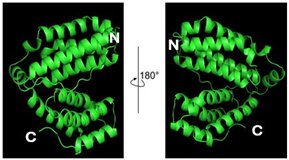	- Fluorescence microscopy [83,84].	- Variable-binding kinetics and affinities depending on the source organism [83].	- High affinity, and compatibility with live-cell imaging [83,84].

PPX_Δ12/PPBD and CHAD protein structures were modelled using SWISS-MODEL online web portal [86,87,88], and visualised with PyMOL Molecular Graphing System, version 3.0 Schrödinger, LLC, New York, NY, USA.

**Table 3:** Methodological comparison of phosphomolybdate assays and end-labelling and functionalisation for polyP quantification.

Method	Applications	Limitations	Advantages
**Malachite Green**	- Spectrophotometric quantification of free orthophosphate following polymer hydrolysis, measured via green complex formation 620–660 nm [91,92].	- Cannot directly distinguish intact polyP polymers, specific chain lengths, or subcellular localisation [97].	- Simple, low-cost, and high analytical sensitivity [89,90].
**Molybdenum Blue**	- Spectrophotometric quantification of reduced phosphomolybdate complexes at 700–890 nm following polyP chemical or enzymatic breakdown [100].	- Requires preliminary acid or enzymatic hydrolysis because polyP cannot be directly detected by the Molybdenum Blue reagent [101]. - Subject to cross-reactivity with silicates, arsenates, and other phosphate-containing compounds, leading to phosphate overestimation [104].	- Inexpensive and highly robust [99].
**End-labelling and Functionalisation**	- Chemical end-labelling with 4-nitrophenol (chromogenic, NOL-polyP) or 4-methylumbelliferone (fluorogenic, MU-polyP) at the terminal phosphates, enabling real-time detection of exo- and endopolyphosphatase activity, biotin/fluorophore tagging for tracking, surface immobilisation, and binding assays [105,108].	- The application of these end-labelled fluorogenic substrates remain challenging due to technically demanding synthesis, variable labelling efficiencies, and dependence on polyP chain length [108].	- Inhibition polyP hydrolysis and analysis of polyP interactions [105].

**Table 4:** Characteristics of advanced biophysical techniques for polyP structure determination.

Method	Limitations	Advantages
**^31^P Nuclear Magnetic Resonance** **(^31^P NMR) spectroscopy**	- Low sensitivity; requires specialised instrumentation [109,110,111,112,113,115,117]. - Measures average chain lengths rather than single chains [109]. - Insoluble/granulated polyP is NMR-invisible [71].	- Bulk chain-length determination [109,110,111]. - Intracellular and in vivo phosphometabolic profiling [113,117].
**Chromatography** **(Anion-Exchange/Size-Exclusion HPLC)**	- Strictly an in vitro method requiring destructive extraction/purification workflows [113,117]. - Vulnerable to stationary-phase matrix column artefacts with long chains [122,123,124].	- High-resolution separation of phosphate mixtures [118,119,120,121,122].
**Mass spectrometry**	- Direct ESI-MS characterisation has only been demonstrated for short-chain polyP (up to tetrapolyphosphate); quantifying polyP requires indirect enzymatic digestion steps [128,130].	- Mass spectrometry provides critical insights into polyP protein dynamics [127].
**FTIR**	- Low mass sensitivity and high spectral background overlap with other biological phosphate fractions [131].	- Allows non-destructive label-free detection of intracellular phosphate-containing compounds [129,130,131].

**Table 5:** Preferred method and essential controls for polyP detection.

Question	Preferred Method	Essential Control
**Is polyP present?**	DAPI/TBO gel, PPBD/CHAD staining	PPX treatment
**Where is polyP located?**	JC-D7/JC-D8, PPBD/CHAD microscopy	RNase/DNase, PPX, competition with polyP
**How much polyP is present?**	Hydrolysis + Malachite Green/Molybdenum Blue	free Pi subtraction, extraction recovery
**What is the chain length?**	PAGE, HPLC, ^31^P NMR	synthetic polyP standards
**Which proteins interact with polyP?**	affinity enrichment + LC-MS/MS	polyP competition, salt controls

## Data Availability

The original contributions presented in this study are included in the article. Further inquiries can be directed to the corresponding author.
